# Quantitative evaluation of an outreach case management model of care for urban Aboriginal and Torres Strait Islander adults living with complex chronic disease: a longitudinal study

**DOI:** 10.1186/s12913-020-05749-7

**Published:** 2020-10-06

**Authors:** Deborah A. Askew, Samantha J. Togni, Sonya Egert, Lynne Rogers, Nichola Potter, Noel E. Hayman, Alan Cass, Alex D. H. Brown, Philip J. Schluter

**Affiliations:** 1 The University of Queensland, Primary Care Clinical Unit, Royal Brisbane and Women’s Hospital, Brisbane, Queensland 4029 Australia; 2 Southern Queensland Centre of Excellence in Aboriginal and Torres Strait Islander Primary Health Care, Queensland Health, Wirraway Pde, Inala, Queensland 4077 Australia; 3 Menzies School of Health Research, Charles Darwin University, Darwin, Australia; 4 South Australian Health and Medical Research Institute, North Terrace, Adelaide, Australia; 5 University of Adelaide, North Terrace, Adelaide, Australia; 6 University of Canterbury - Te Whare Wānanga o Waitaha, School of Health Sciences, Christchurch, New Zealand

**Keywords:** Aboriginal and Torres Strait Islander, Chronic disease, Outreach case management, Primary health care, Indigenous Australian

## Abstract

**Background:**

Chronic diseases are the leading contributor to the excess morbidity and mortality burden experienced by Aboriginal and Torres Strait Islander (hereafter, respectfully, Indigenous) people, compared to their non-Indigenous counterparts. The Home-based Outreach case Management of chronic disease Exploratory (HOME) Study provided person-centred, multidisciplinary care for Indigenous people with chronic disease. This model of care, aligned to Indigenous peoples’ conceptions of health and wellbeing, was integrated within an urban Indigenous primary health care service. We aimed to determine the impact of this model of care on participants’ health and wellbeing at 12 months.

**Methods:**

HOME Study participants were Indigenous, regular patients of the primary health care service, with a diagnosis of at least one chronic disease, and complex health and social care needs. Data were collected directly from participants and from their medical records at baseline, and 3, 6 and 12 months thereafter. Variables included self-rated health status, depression, utilisation of health services, and key clinical outcomes. Participants’ baseline characteristics were described using frequencies and percentages. Generalized estimating equation (GEE) models were employed to evaluate participant attrition and changes in outcome measures over time.

**Results:**

60 participants were enrolled into the study and 37 (62%) completed the 12-month assessment. After receiving outreach case management for 12 months, 73% of participants had good, very good or excellent self-rated health status compared with 33% at baseline (*p* < 0.001) and 19% of participants had depression compared with 44% at baseline (*p* = 0.03). Significant increases in appointments with allied health professionals (*p* < 0.001) and medical specialists other than general practitioners (*p* = 0.001) were observed at 12-months compared with baseline rates. Mean systolic blood pressure decreased over time (*p* = 0.02), but there were no significant changes in mean HbA1c, body mass index, or diastolic blood pressure.

**Conclusions:**

The HOME Study model of care was predicated on a holistic conception of health and aimed to address participants’ health and social care needs. The positive changes in self-rated health and rates of depression evinced that this aim was met, and that participants received the necessary care to support and improve their health and wellbeing.

## Background

Since 2009, the Australian Prime Minister has addressed the nation’s parliament each year on progress towards improving health outcomes for Aboriginal and Torres Strait Islander people (hereafter, respectfully, Indigenous). Of particular concern is the reduced life expectancy of Indigenous people compared to their non-Indigenous counterparts. In 2019, the Prime Minister reported that the life expectancy of Indigenous males born between 2015 and 2017 was 8.6 years less than their non-Indigenous counterparts (71.6 years compared with 80.2 years). For Indigenous females, the life expectancy gap was 7.8 years (75.6 years compared with 83.4 years) [1]. This life-expectancy gap is evidence of one of Australia’s most enduring equity, equality and social justice divides [2].

Chronic diseases (CD) are the leading contributor to the excess morbidity and mortality experienced by Indigenous people compared to their non-Indigenous counterparts, and in Australia, account for 64% of the total burden of disease [3]. Additionally, at least 34% of the gap in health outcomes can be attributed to inequalities in education, employment status, overcrowding and household income [3]. These, and the other social determinants of health, are the ‘conditions in which people are born, grow, live, work and age’ [4] – the ‘causes of the causes’ [5]. The inequalities in health status observed in Australia are directly linked to Indigenous peoples’ reduced access to economic and educational opportunities, limited physical infrastructure, and poorer social conditions, compared to their non-Indigenous counterparts [6].

Internationally, CD care has typically focussed on individualistic, compartmentalized and biomedical interventions that are largely antithetical to Indigenous peoples’ understandings and conceptions of health [7, 8]. Here, health is perceived as a holistic and collective concept that encompasses the social, emotional and cultural wellbeing of the whole community. Strategies to improve Indigenous people’s health need to address the broad determinants of Indigenous health, and improve access to comprehensive, holistic, culturally informed, community-based health care [8]. Underpinned by these understandings of health and health care, a holistic model of person-centred CD case management was developed and implemented in an urban Indigenous primary health care service [9]. The model of care privileged Indigenous people’s understandings of health and aimed to give people agency in decisions about the focus and methods of their own health care. Evaluation of this model of care through the Home-based, Outreach case Management of chronic disease Exploratory (HOME) Study identified that this model of care was feasible, acceptable and appropriate and thus it became a permanent component of the armamentarium of CD care at the primary health care service [9]. This enabled recruitment of additional participants and longer term follow up to determine if the positive outcomes were sustained. Quantitative and qualitative data were collected, with the latter aiming to understand the key features of value of the model of care and will be reported elsewhere. This paper reports the longitudinal analysis of selected quantitative data to determine the impact of this model of care on participants’ self-rated health status, mental health, health service utilisation and biomedical health outcomes after 12 months of case management support.

## Methods

### Study design and setting

Details of the HOME Study design and model of care have been reported elsewhere [9] and are summarised here. The HOME Study was implemented at the Southern Queensland Centre of Excellence in Aboriginal and Torres Strait Islander Primary Health Care (COE), a Queensland Government health service located in Inala (a south-western suburb of Brisbane) that provides primary health care predominantly to Indigenous people [10].

### Participants

To be eligible, participants had to: self-identify as Aboriginal and/or Torres Strait Islander; attend the COE at least twice in the previous 2 years; live within a 1-h drive of the COE; have a confirmed diagnosis of type 2 diabetes (T2D), cardiovascular disease (CVD) (including congestive cardiac failure or a history of coronary artery disease or stroke), chronic respiratory disease (including asthma or chronic obstructive pulmonary disease), or chronic kidney disease (CKD) defined as eGFR between 15 and 60 mL/min/1.73m^2^; have complex health and/or social care needs; and be able to provide informed consent [9]. Recognising that older people attending the Inala COE were more likely to have lower levels of health literacy and higher levels of CD than their younger counterparts [11, 12], verbal explanations of the study were provided to potential participants by their general practitioner (GP) and by one of the registered nurse case managers (CMs). Potential participants were also provided written information about the study, and were accorded the opportunity to decide, in their own time, whether or not to participate [9].

Patients were ineligible if they were pregnant; had end-stage renal failure and/or receiving renal dialysis; had limited life expectancy; required extensive support in daily life; were an aged care facility resident, or were incarcerated at the time of recruitment. Eligibility was reassessed throughout the study, and participants who became ineligible were withdrawn from the study [9].

### HOME study model of care

The HOME Study model of care had two phases [9]. Phase one aimed to identify what each participant needed to be well and healthy, and phase two aimed to ensure that participants’ needs were met by the health and social care systems. Importantly, participants defined their own health needs based on their own conceptions of health, thus privileging Indigenous conceptions of health [9].

In phase one, the CMs completed a compressive needs assessment which included a retrospective review of each participant’s electronic medical records (according to established audit protocols [13]) and an assessment conducted in participants’ homes. The former focussed on past medical history and health service utilisation and the latter focussed on the participant as a person: their cultural heritage, their family, their psychosocial status, and their health and wellbeing needs and goals. Participants were encouraged to identify goals that were important to them, rather than goals that reflected conversations they had previously had with health professionals [9]. Results of the needs assessment were presented at a multidisciplinary case conference, attended by the participant and family members (if they wished), their CM, and their GP. COE allied health staff (dietitian, psychologist or social worker) involved in the participant’s care also attended. Importantly, the CMs ensured that participants’ health and wellbeing goals were centred in case conference discussions and outcomes. A comprehensive wellbeing plan was then collaboratively developed by the participant and CM that clearly identified the actions and people necessary to achieve the participants’ goals [9].

Phase two focused on implementation of the wellbeing plan [9]. The CMs facilitated progress towards goal attainment, provided a point of reference and advocacy for participants navigating the health and social care systems, and encouraged and empowered participants to be active members of their health care teams. Thus, the CMs’ role was one of care-coordination and advocacy, rather than provision of any clinical nursing [9].

### Instruments

Chart audits and home assessments were completed at baseline, and approximately 3, 6 and 12 months thereafter to gain a holistic understanding of each participant, and their health and social care needs. Socio-demographic variables assessed at baseline included self-identified ethnicity; identification of own, maternal and paternal traditional Country or clan group; experiences of forced removal from traditional Country or family; connection to, and strengths of family and community; and self-reported date of birth, annual income and highest level of education. The CMs used a visual tool to hold a guided conversation with participants about what kept them healthy and strong, and what took their strength away. The tool had a variety of illustrations including pictures of family, activities such as fishing or sport, and cultural activities such as dancing, as well as pictures of housing, medicines, food, alcohol, cigarettes, etc. For example, having family nearby might provide physical and psychological support for some participants, thus keeping them healthy and strong. Conversely, family might also take away participants’ strength through conflict or ill-health. Self-reported health status was assessed using a single-item measure where participants self-rated their current health status on a five-point scale from excellent to poor [14]. Depression was assessed using the culturally appropriate adapted 9-item Patient Health Questionnaire (aPHQ-9) [15]. Measurement of health services utilisation at baseline included the number of visits to allied health professionals in the previous 12 months; the number of referrals to medical specialists made and attended in the previous 12 months; and the number of emergency department attendances and hospital admissions in the previous 2 years. At subsequent measurement waves, the timeframe for these variables was the time since the previous assessment. Key clinical outcomes included HbA1c, body mass index (BMI), and blood pressure (BP). Table 1 presents the schedule and broad content of the assessments.

**Table 1:** Schedule and content of assessments for HOME Study participants

	Baseline	3-months	6-months	12-months
*Variables assessed from medical chart audit*				
Chronic disease diagnoses*	✓	✓	✓	✓
HbA1c (%)	✓	✓	✓	✓
Body Mass Index (kg/m^2^)	✓	✓	✓	✓
Blood pressure (mmHg)	✓	✓	✓	✓
Health services utilisation	✓	✓	✓	✓
Medications	✓	✓	✓	✓
*Variables assessed during home assessments*				
Socio-demographics	✓			
Depression^a^	✓		✓	✓
Social and emotional wellbeing	✓		✓	✓
Self-rated health status^b^	✓		✓	✓
Lifestyle factors	✓	✓	✓	✓
Medication use and medication literacy	✓	✓	✓	✓

* At baseline, current diagnoses of chronic diseases were recorded. New diagnoses were recorded at subsequent assessments;^a^Depression assessed using adapted PHQ-9 – dichotomised as moderate to severe depression vs. otherwise; ^b^Self-rated health status dichotomised as good, very good or excellent vs. poor or fair;

### Statistical analysis

Reporting of analyses was informed by the Strengthening the Reporting of Observational Studies in Epidemiology (STROBE) guidelines [16]. Frequencies and percentages were used to describe the baseline characteristics of participants over time, and the patterns of missing values were evaluated using binomial generalized estimating equation (GEE) models with an unstructured correlation matrix and robust Huber-White sandwich variance estimators. Self-rated health was dichotomised by grouping “good”, “very good” and “excellent” responses into one category, and “poor” and “fair” responses into another. Responses to the depression screen were dichotomised by grouping “severe” and “moderate” levels of depression into one group, and “mild” and “no” depression into another. Similarly characterised binomial GEE models were used to assess changes in these binary variables over time. Binomial, normal, and negative binomial GEE models were also employed for the analysis of the remaining binary, scale, and count variables, respectively. Negative binomial GEE models were adopted for the health service utilisation assessed from medical chart audits as all counts were dispersed. In these models, exposure was included and defined to account for the different time intervals of the trial. Counts at baseline were calculated over the preceding 24 months for hospitalisations and 12 months for appointments with allied health professionals or medical specialists other than GPs. Counts at 3- and 6-months used a 3 months exposure, while counts at 12-months used a 6 months exposure. Statistical significance was assessed via Wald’s Type III statistic. All analyses were performed using SAS version 9.3 (SAS Institute Inc., Cary, NC, USA), and α = 0.05 was used to define significance for all tests.

### Community support, ethical approval and consent to participate

Community support for the HOME Study was provided by the Inala Community Jury for Aboriginal and Torres Strait Islander Health Research and ethical clearance was obtained from the Metro South Human Research Ethics Committee (reference number: HREC/12/QPAH/294). All participants provided written informed consent prior to any data collection [9].

## Results

### Participants

**Fig. 1:**
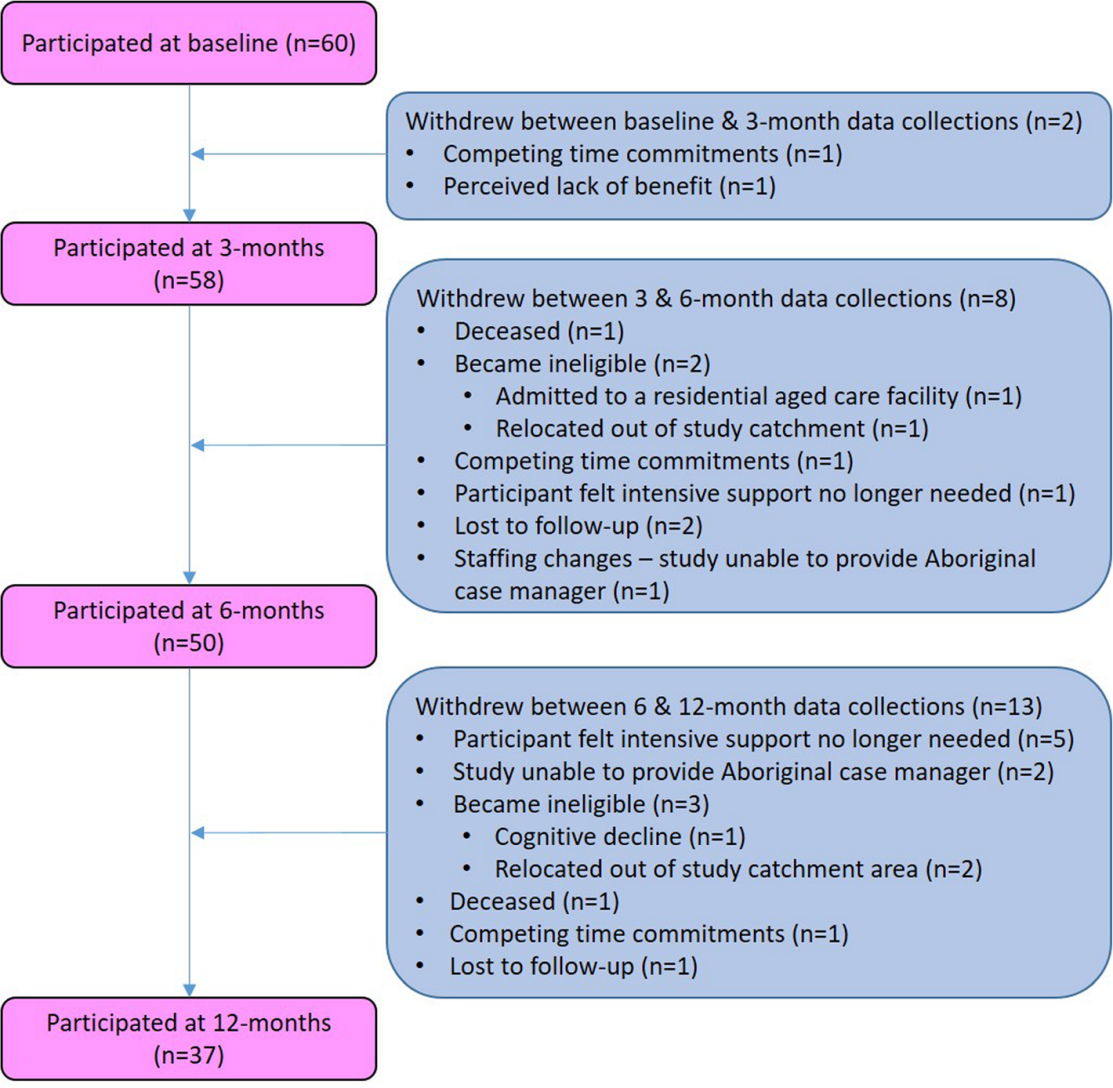
HOME Study Participant flow

The receipt of recurrent funding enabled the HOME Study model of care to become a permanent program at the COE, and recruitment to this research study continued for a further 12 months. Included here are 37 people who had completed 6 month follow-up in the initial period of this study [9] and an additional 23 newly recruited participants. Thus, a total of 60 participants were included at baseline, with 58 (97%) participating at 3-months, 50 (83%) at 6-months, and 37 (62%) participants completing the 12-month assessment (Fig. 1). The most common reasons for withdrawing included the participant determining they no longer required the intensive support provided by the CMs (6/23), or they became ineligible (cognitive decline or relocation out of study area) (5/23).

At baseline, 53 (88%) identified as being Aboriginal only, 1 (2%) as Torres Strait Islander only, and 6 (10%) as both Aboriginal and Torres Strait Islander. At this initial assessment, 62% of participants were women, 34% were aged ≥65 years, 50% had an annual income of <AUD$20,800, and primary schooling was the highest level of educational attainment for 47%; see Table 2. Table 2 also provides the distribution of measured baseline socio-demographics over time. From binomial GEE analyses, there was no differential attrition over time by sex (*p* = 0.65) or age category (*p* = 0.44), although differential attrition was observed for participants’ annual income (*p* = 0.03) and their highest level of educational attainment (*p* = 0.03). Those with annual income less than AUD$20,800 were relatively more likely to withdraw, as were those with post-secondary education. Once again, using binomial GEE analyses, accounting for time and employing an unstructured covariance matrix, there was also no differential attrition by baseline measurement of depression (*p* = 0.92), self-rated health status (*p* = 0.11), or multi-morbidity (*p* = 0.09).

**Table 2:** Distribution of baseline characteristics of study participants at baseline (*n* = 60), 3-months (*n* = 58), 6-months (*n* = 50), and 12-months (*n* = 37)

	Baseline		3-months		6-months		12-months	
	n	(%)	n	(%)	n	(%)	n	(%)
*Sex*								
Male	23	(38)	22	(38)	19	(38)	16	(43)
Female	37	(62)	36	(62)	31	(62)	21	(57)
*Age (years)*^a^								
< 55	10	(17)	9	(16)	7	(14)	7	(19)
55–59	11	(19)	11	(19)	8	(16)	4	(11)
60–64	18	(31)	17	(30)	17	(34)	12	(33)
65+	20	(34)	20	(35)	18	(36)	13	(36)
*Annual income*^*b*^								
< $20,800	30	(50)	30	(52)	24	(48)	16	(43)
$20,800–$31,199	8	(13)	8	(14)	8	(16)	8	(22)
$31,200+	1	(2)	1	(2)	1	(2)	1	(3)
Unknown	21	(35)	19	(33)	17	(34)	12	(32)
*Highest level of education attained*^c^								
Primary	27	(47)	27	(47)	25	(50)	19	(51)
Secondary	15	(26)	15	(26)	13	(26)	13	(35)
Post-secondary	16	(28)	16	(28)	12	(24)	5	(14)

^a^missing value for 1 participant; ^b^Income in Australian dollars: categories reflect those used by the Australian Bureau of Statistics in the 2011 Census; ^c^missing values for 2 participants

### Chronic diseases

At baseline, 49 (86%) participants had T2D, 24 (40%) had CVD, 31 (52%) had chronic respiratory conditions, and 12 (20%) participants suffered from CKD; see Table 3. Four participants (8%) had four CDs, 15 (28%) had three, 18 (34%) had two, and 16 (30%) had one CD. Binomial GEE analyses failed to detect change in any of these rates over time (all *p* > 0.05).

**Table 3:** Chronic disease status profile at baseline (*n* = 60), 3-months (*n* = 58), 6-months (*n* = 50), and 12-months (*n* = 37)

	Baseline		3-months		6-months		12-months	
	n	(%)	n	(%)	n	(%)	n	(%)
*Type 2 diabetes*								
Yes	49	(86)	47	(84)	42	(86)	28	(82)
No	8	(14)	9	(16)	7	(14)	6	(18)
*Cardiovascular disease*								
Yes	24	(40)	22	(38)	20	(41)	14	(40)
No	36	(60)	36	(62)	29	(59)	21	(60)
*Chronic respiratory conditions*								
Yes	31	(52)	29	(51)	25	(51)	17	(47)
No	29	(48)	28	(49)	24	(49)	19	(53)
*Chronic kidney disease*								
Yes	12	(20)	12	(21)	13	(26)	9	(26)
No	48	(80)	46	(79)	37	(74)	25	(74)
*Multi-morbidity (≥2 chronic diseases)*								
Yes	37	(70)	34	(65)	33	(70)	22	(69)
No	16	(30)	18	(35)	14	(30)	10	(31)

### Self-rated health status

At baseline, only 1 (2%) participant rated their health status as excellent, 2 (4%) as very good, and 16 (28%) as good, whereas 27 (47%) rated their health as fair, and 11 (19%) as poor. Table 4 presents the dichotomised self-rated health status variable distribution over time. From the binomial GEE model, participants were significantly more likely to self-report “good”, “very good” or “excellent” health status at 6-months (*p* = 0.03) and 12-months (*p* < 0.001) compared to baseline.

**Table 4:** Distribution of self-rated health assessed during home assessments at baseline (*n* = 60), 6-months (*n* = 50), and 12-months (*n* = 37)

*Self-rated health status*	Baseline		6-months		12-months	
	n	(%)	n	(%)	n	(%)
Good, very good, excellent	19	(33)	26	(53)	22	(73)
Poor or fair	38	(67)	23	(47)	8	(27)

Note: 3 (5%) observations missing at baseline, 1 (2%) at 6-months, and 7 (19%) at 1-year

Concomitantly, rates of depression decreased over the assessment periods, from 44% (25/57) at baseline, to 33% (16/49, *p* = 0.19) at 6-months, and 19% (5/27, *p* = 0.03) at 12-months. Taken together, participant’s self-reported holistic conceptions of their health significantly improved after 12 months despite living with complex CD.

### Health service utilisation and biomedical health outcomes

Table 5 includes the observed rates of health service utilisation and distribution of key clinical outcome variables assessed from medical chart audits at baseline, 3-, 6-, and 12-months. From the negative binomial GEE analyses, rates of appointments with allied health professionals significantly increased over time (*p* < 0.001) with higher rates observed at each measurement wave compared to baseline; as did the rates of referrals to medical specialists other than GPs (*p* = 0.001). However, there were no observed changes in the rates of hospital emergency department attendance (*p* = 0.63) or hospitalisations (*p* = 0.92) over time, nor in the proportion of specialist appointments that were attended (*p* = 0.29).

**Table 5:** Rates of health service utilisation from medical chart audits and distribution of key clinical outcome variables assessed from medical chart audits at baseline (*n* = 60), 3-months (*n* = 58), 6-months (*n* = 50), and 1-year (*n* = 37)

*Health service utilisation*	Baseline		3-months		6-months		1-year	
	n (%)	rate/mth (SD)	n (%)	rate/mth (SD)	n (%)	rate/mth (SD)	n (%)	rate/mth (SD)
Allied health visits^a^	60 (100)	0.15 (0.26)	58 (100)	0.45 (0.62)	50 (100)	0.48 (0.46)	36 (97)	0.57 (1.11)
Medical specialist referrals^a^	60 (100)	0.14 (0.12)	58 (100)	0.26 (0.30)	50 (100)	0.25 (0.30)	37 (100)	0.20 (0.20)
Proportion of referrals attended^b^	46 (100)	0.74 (0.38)	28 (97)	0.88 (0.32)	24 (100)	0.75 (0.44)	26 (100)	0.78 (0.41)
Emergency department presentations^a^	57 (95)	0.03 (0.05)	55 (95)	0.03 (0.12)	48 (96)	0.01 (0.07)	36 (97)	0.02 (0.07)
Hospitalisations^a^	58 (97)	0.09 (0.14)	55 (95)	0.09 (0.19)	46 (92)	0.12 (0.35)	36 (97)	0.09 (0.17)
*Key clinical outcomes*	n (%)	mean (SD)	n (%)	mean (SD)	n (%)	mean (SD)	n (%)	mean (SD)
HbA1c (%)^a^	49 (82)	7.9 (1.9)	39 (67)	7.9 (1.8)	22 (44)	7.8 (1.9)	28 (76)	8.0 (1.7)
BMI (kg/m^2^) ^a^	59 (98)	34.5 (8.8)	45 (78)	35.4 (10.6)	26 (52)	36.6 (8.7)	32 (86)	35.3 (8.4)
Systolic blood pressure (mmHg) ^a^	56 (93)	134 (20)	50 (86)	127 (16)	37 (74)	127 (19)	37 (100)	126 (22)
Diastolic blood pressure(mmHg) ^a^	56 (93)	77 (9)	51 (88)	76 (11)	37 (74)	76 (9)	37 (100)	78 (14)

^a^ n represents the number of participants where complete information was available for each variable and percentages are calculated using the number of participants in the study at each measurement point as the denominator

^b^ n gives the number of participants where complete information on attendances for every medical specialist referral was recorded and percentages are calculated using the number of medical specialist referrals as the denominator

In terms of key biomedical outcomes, there was an overall significant decrease in mean systolic BP over time (*p* = 0.02), but no overall significant changes in mean HbA1c (*p* = 0.89), mean BMI (*p* = 0.80), or mean diastolic BP (*p* = 0.86) over time; see Table 5.

## Discussion

The HOME Study model of holistic, person-centred outreach case management has already been demonstrated as being feasible, acceptable and appropriate for Indigenous people with complex CD and their primary health care service [9]. However, the question remained as to whether it delivered a sustained positive impact on Indigenous people’s health and wellbeing. The results reported here demonstrate significant improvements in participants’ own conceptions of their health with rates of depression decreasing from nearly half of participants at baseline to one in five participants at 12-months. Similarly, self-rated health status improved, from about one third rating their health as good, very good or excellent at baseline to about three quarters rating their health thus at 12-months. With the exception of systolic BP, where a reduction was observed over the 12-months, there were no significant changes in the other clinical outcomes measured. Access to allied health professionals and medical specialists other than GPs increased, although rates of hospitalisations and presentations to emergency departments did not change. Contextualising these results, study participants all had complex CD and had health or social care needs requiring intensive support and care coordination. That self-rated health status and depression improved and objective biomedical measures did not decline suggests that our model of care successfully identified and addressed participants’ health and social care needs.

Outreach case management, as operationalised through the HOME Study model of care, was a collaborative and person-centred model of care. In contrast to many interventions and initiatives aiming to improve the health of Indigenous peoples by focusing on behavioural risk factors, the HOME Study used a participatory approach whereby participants set their own health and wellbeing goals, based on their own priorities and definitions of health and wellbeing. The resulting improvements in self-rated health status and improvements in the rates of depression are testament to appropriateness of this approach. Similar outcomes have been reported in other countries, where meaningful and successful chronic disease management programs encompass the physical, emotional, intellectual, spiritual and cultural foundations of Indigenous peoples’ conceptions of health and wellbeing [17]. Some may argue that the lack of improvement in clinical outcomes are evidence of a weakness in this model of care. However, self-rated health status is a predictor of future morbidity and mortality [18], and comorbid depression incrementally worsens health status in people with CD and may also impact on people’s capacity for self-management [19, 20]. Thus, these improvements in participants’ health status are socially and clinically important.

CDs are long lasting and progressive conditions with persistent effects that inflict considerable psychological and physiological burden on those affected [21]. Therefore, while it is possible that, for some participants, changes in life’s circumstances may have resulted in improvements in their mental and self-rated health status over time, it is unlikely that the magnitude of improvements would have occurred without the HOME Study model of care. Reflection on the fundamental principles underpinning the model of care provides some clues as to why these improvements in health status were observed. The model of care was co-developed with input from participants and their primary health care service rather than being imposed by external parties. The CMs were respectful of participants’ need for self-determination over their health care and ensured that power and control remained with participants. The CMs also provided positive social interactions, tangible social support to participants [22] – they coordinated participants’ care, they advocated for participants with health and social care agencies to organise services, they arranged transport for participants to attend appointments, and they acted as ‘boundary spanners’ by forging links and connections across service boundaries to ensure effective coordination of participants’ care needs [23]. The fragmented and suboptimal care frequently experienced by people living with multi-morbidities and complex social care needs is well documented [24]. The co-location and integration of the HOME Study model of care within an Indigenous primary health care service minimised the risk of fragmentation because the CMs and the primary health care professionals worked as a team to provide holistic, person-centred, coordinated care.

The embodiment of the holistic Aboriginal definition of health [7] in the model of care and the key outcome measure of self-rated health status was a key strength of this study. Participants were supported to be well and healthy, however they defined this, and inclusion of self-rated health status ensured that participants’ own conceptions of their health status were captured. Additional strengths of the study included the use of routinely collected clinical data to reduce participant burden, in addition to data collected directly from participants, and the use of developmental evaluation [25] to collaboratively develop and adapt the model of care to meet the needs of participants and health service staff [26].

Some of the challenges experienced in conducting this study have been described elsewhere [9]. In brief, a key challenge was the competing priorities of research and health care service delivery. This challenge was managed through the creation of a strong project team, effective communication between the project team and the primary health care service, and the exploratory nature of the study that allowed adaptation of the model of care based on feedback from participants and the primary health care providers. Further limitations of this study relate to participant attrition. However, with the exception of baseline income and highest level of education attained, withdrawal from the study was not associated with any sociodemographic or key clinical characteristic. This attrition may affect the external validity of the findings, although our results do provide understanding of the impact of the model of care under usual and pragmatic conditions [27].

## Conclusion

Given the high rates of CD experienced by Indigenous Australians, and the associated negative impact on health and wellbeing, innovative models of care that align with Indigenous conceptualisations of health are needed. While outreach case-management is not a novel approach to CD care, the HOME Study model of care focus of meeting participants’ health and wellbeing goals ensured that each participant’s unique needs were met, rather than a one-size-fits-all approach. The model of care was functionally and philosophically integrated into the primary health care service and expanded the capacity of the primary health care service to provide comprehensive, continuous and coordinated care – the hallmarks of primary health care [28]. The feasibility, acceptability and appropriateness of this model of care has been reported elsewhere [9], and here, the sustained positive impact on participants’ health and wellbeing has been established. Further research is needed to understand the core features of value of the model of care to enable scaling up and spreading to other contexts to reshape how CD care for Indigenous Australians is conceptualised and delivered, and replication and adaptation of the model of care elsewhere is needed before generalisability can be confidently asserted.

## Data Availability

The data that support the findings of this study are not publically available, but are available from the COE, via the corresponding author, providing appropriate ethical and community approvals are obtained.

## Abbreviations

aPHQ-9: Adapted Patient Health Questionnaire 9 items
BMI: Body mass index
BP: Blood pressure
CD: Chronic Disease
CKD: Chronic Kidney Disease
CM: Case manager
COE: Southern Queensland Centre of Excellence in Aboriginal and Torres Strait Islander Primary Health Care
CVD: Cardiovascular Disease
GEE: Generalised Estimating Eq.
GP: General Practitioner
HOME Study: Home-based, Outreach case Management of chronic disease Exploratory Study
T2D: Type 2 Diabetes
